# Kinetic analysis of mechanoradical formation during the mechanolysis of dextran and glycogen

**DOI:** 10.3762/bjoc.13.116

**Published:** 2017-06-19

**Authors:** Naoki Doi, Yasushi Sasai, Yukinori Yamauchi, Tetsuo Adachi, Masayuki Kuzuya, Shin-ichi Kondo

**Affiliations:** 1 Laboratory of Pharmaceutical Physical Chemistry, Gifu Pharmaceutical University, 1-25-4 Daigaku-Nishi, Gifu 501-1196, Japan; 2 Department of Pharmaceutical Physical Chemistry, Faculty of Pharmaceutical Sciences, Matsuyama University, 4-2 Bunkyo-cho, Matsuyama, Ehime 790-8578, Japan; 3 Laboratory of Clinical Pharmaceutics, Gifu Pharmaceutical University, 1-25-4 Daigaku-Nishi, Gifu 501-1196, Japan; 4 Department of Health and Welfare, Faculty of Human Welfare, Chubu Gakuin University, 2-1 Kirigaoka, Seki-shi, Gifu 501-3993, Japan

**Keywords:** dextran, electron spin resonance (ESR), glycogen, mechanoradical, polysaccharide

## Abstract

A detailed electron spin resonance (ESR) analysis of mechanically induced free radicals (mechanoradicals) formation of glucose-based polysaccharides, dextran (Dx) and glycogen (Gly) was performed in comparison with amylose mechanoradicals. The ESR spectra of the samples mechanically fractured at room temperature were multicomponent. The radical concentration of Dx and Gly mechanoradicals gradually decreased during vibratory milling after reaching the maximum value. Although the molecular weight of Dx or the particle diameter of Gly steeply diminished until reaching the each maximum value of radical concentration, after that the molecular weight or the particle diameter slowly decreased. These results suggested that Dx and Gly mechanoradicals might be more unstable than amylose radicals possessing an intramolecular helical structure due to the branched structure.

## Introduction

**Figure 1:**
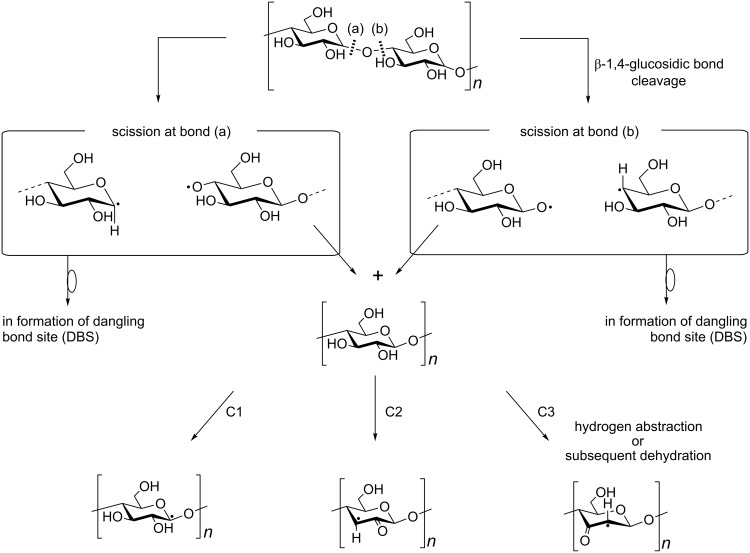
Structures of discrete mechanoradicals and the reaction sequence for their formation from cellulose [5].

There are many reports on the mechanolysis of synthetic and natural polymers. It is well-known that mechanically induced radicals, so-called mechanoradicals, are produced by the mechanolysis of a polymer at a temperature below its glass-transition temperature (*T*_g_) due to the disruption of the polymer main chain [1]. Although most pulverization operations for a practical use are carried out at room temperature, electron spin resonance (ESR) spectroscopy analyses of mechanoradical formation have generally been conducted at low temperature (77 K) [2]. In previous papers we discussed the mechanoradical formation through mechanolysis of synthetic polymers [3–4] and polysaccharides such as amylose and cellulose [5] at room temperature under strictly anaerobic conditions. ESR spectral analysis and the progressive changes in the physicochemical properties were also studied in detail. As a representative example, Figure 1 shows the radical structures observed following mechanolysis of cellulose and summarizes the possible reaction sequence.

The cellulose mechanoradicals, which were generated by subsequent radical reactions such as hydrogen abstraction and/or recombination after polymer main-chain scission, could be assigned to alkoxylalkyl-type radicals at the C1 and acylalkyl-type radicals at the C2 and/or C3 positions. Therefore, these observed mechanoradicals were mid-chain radicals.

**Figure 2:**
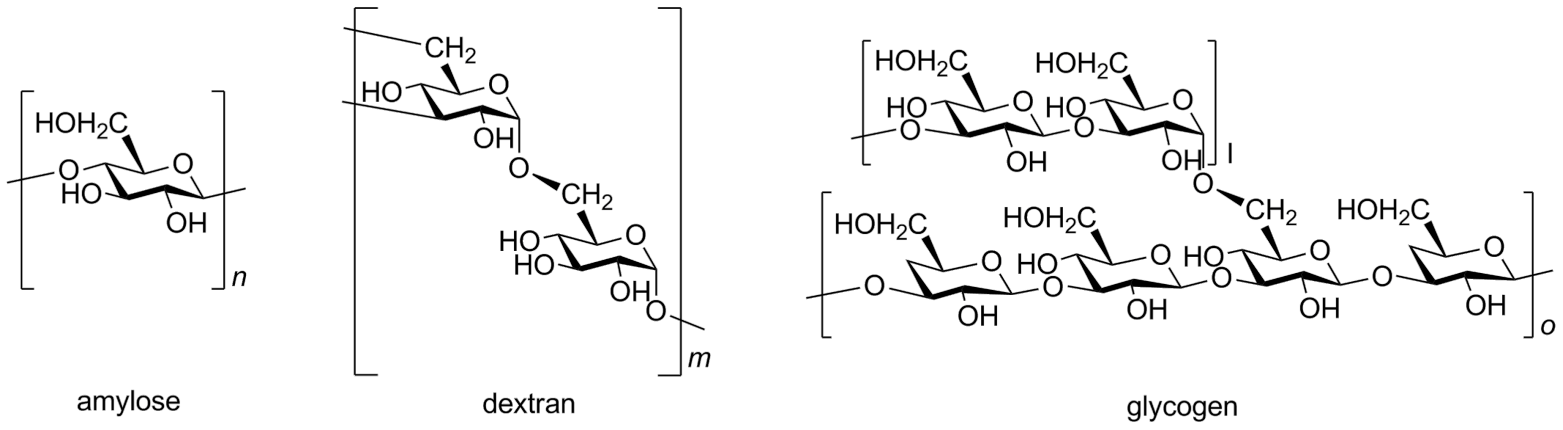
Schematic structure of amylose, dextran and glycogen.

Great attention has been paid to graft polymerization of synthetic polymers onto polysaccharides, because this method easily produces a polymer combining the advantages of both natural and synthetic macromolecules [6]. A polysaccharide possessing functional group on its backbone that allows to initiate the polymerization is frequently used to synthesize such a graft polymer [7]. Dextran (Dx), a biodegradable polysaccharide, has been utilized as a graft copolymer backbone. The glycosidic linkages between the α-glucose units of Dx synthesized from *Leuconostoc mesenteroides* are composed of approximately 95% α-D-1,6-linkages, which form a straight chain, and 5% α-1,3-linkages, from which branches begin, as shown in Figure 2 [8–10].

The grafting of synthetic polymers onto Dx has generally been carried out using oxygen-based radicals produced via a hydrogen abstraction method (e.g., radical initiation, γ-irradiation) from hydroxy groups [11–14]. However, as the polysaccharide backbone is unstable under these harsh and high temperature conditions, these compounds are not suitable for condensation polymerizations to synthesize graft copolymers [15]. In general a radical polymerization can be used for the synthesis of graft polymers consisting of vinyl monomers and polysaccharides [16]. Mid-chain radicals can also be formed by mechanolysis of hydroxyethylcellulose (HEC), so that it was hoped that the mechanolysis of HEC in the presence of vinyl monomers would produce graft copolymers possessing synthetic polymers as branches. Sakaguchi et al. reported a diblock copolymer formation through the mechanochemical reaction of bacterial cellulose and methyl methacrylate in vacuum at 77 K [17]. Solala et al. studied the mechanochemical reaction of cotton in the presence of styrene and disclosed the formation of polystyrene on the cotton [18]. In a previous paper, we reported the synthesis of water-soluble graft polymeric prodrugs through the mechanochemical reaction of HEC and methacryloyl derivatives of 5-fluorouracil [19]. We also discussed the nature of drug release from the polymeric prodrugs produced as a prototype [19]. However, HEC is not metabolized by humans. Therefore if one could use a polymer metabolized by humans, such as Dx or glycogen (Gly), a promising graft polymeric prodrug could be obtained through a mechanochemical reaction in a totally dry process. It is necessary to elucidate the structure and stability of mechanoradicals of Dx and Gly as a pre-screening test for the development of such a graft polymeric prodrug. However, to our knowledge, there are no reports describing the formation of Dx or Gly mechanoradicals at room temperature.

In this paper we discuss the mechanoradical formation from Dx and Gly at room temperature in detail. To obtain fundamental insights into the mechanolysis of Dx and Gly, we conducted detailed ESR spectra analyses of the Dx and Gly mechanoradicals in comparison with those of amylose. Because amylose is an α-glucose-based polysaccharide and its detailed analysis of ESR spectra of mechanoradicals has been studied [5], we selected it as a reference sample.

In a previous paper [5], we studied the radical formation by plasma-irradiation and mechanolysis of amylose and the β-glucose-based linear polysaccharide, cellulose, in view of the difference of bonding type. The present paper focused on the polymer structure, such as helical (amylose), branched (Dx) and hyper-branched structure (Gly), to clarify the stability of component radicals depending on the polymer structure. Progressive changes in Dx molecular weight and Gly particle diameter were also investigated.

## Results and Discussion

**Figure 3:**
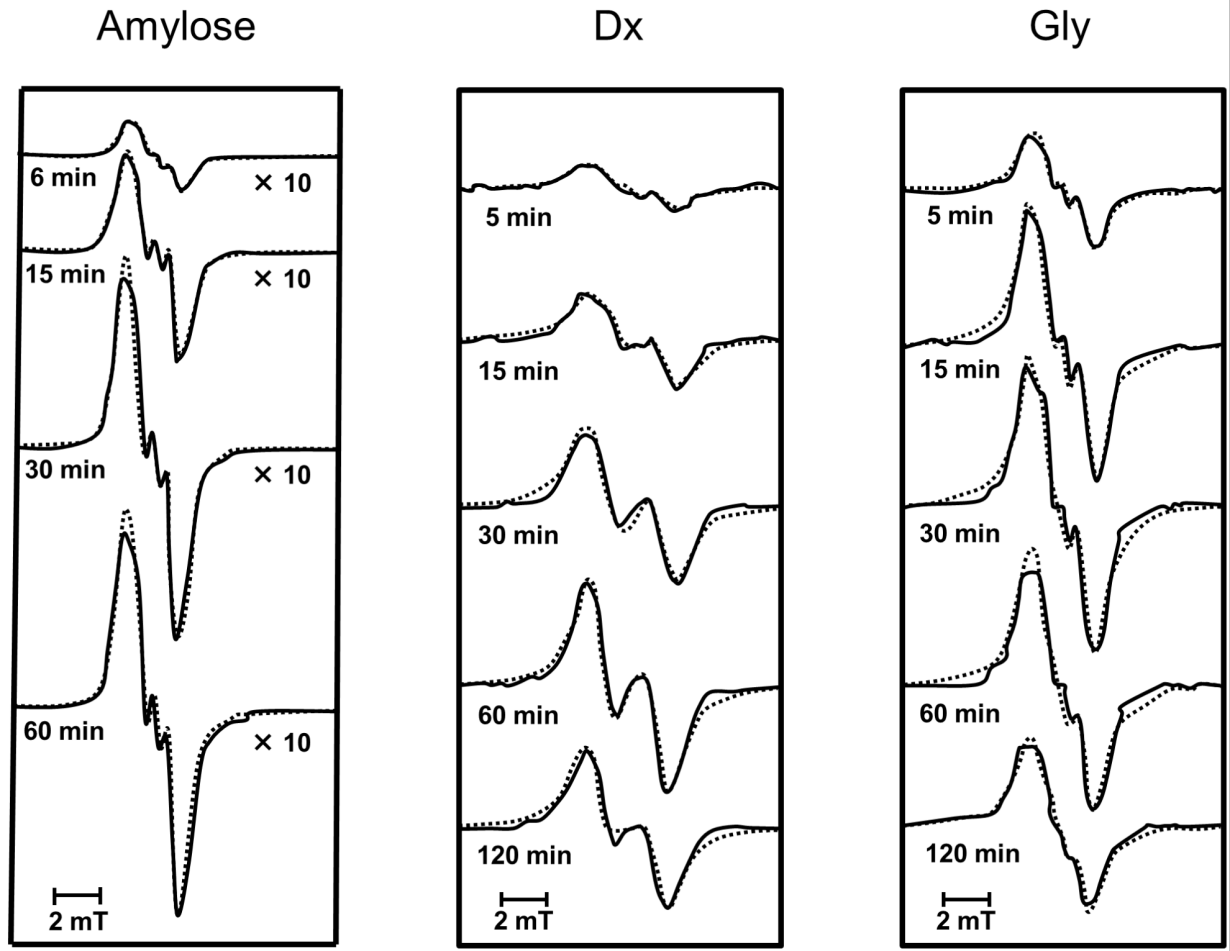
Progressive changes in observed ESR spectra of fractured amylose [5], Dx, and Gly, together with simulated spectra (shown as dotted lines).

Figure 3 shows the progressive changes in the ESR spectra of amylose [5], Dx, and Gly mechanically fractured by vibratory ball milling at 60 Hz at room temperature for various periods of time under anaerobic conditions, together with the corresponding simulated spectra (shown as dotted lines).

It can be seen from Figure 3 that spectra of amylose, Dx, and Gly appreciably differ from one another, but the individual spectra remained nearly unchanged during the course of vibratory milling.

**Figure 4:**
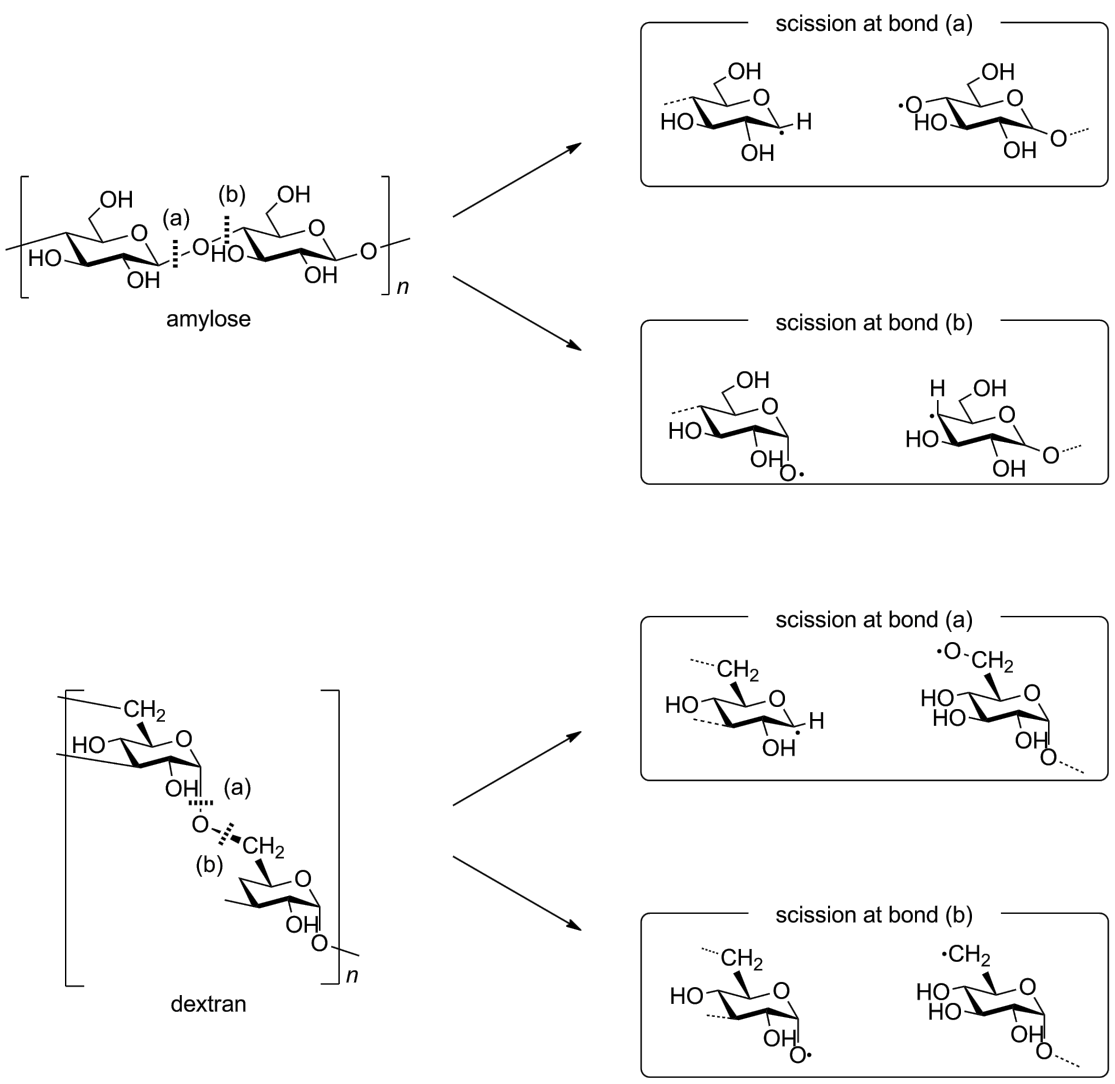
Schematic representation of bond cleavage at α-1,4- and α-1,6-bonds.

As mentioned above, amylose is a linear poly-D-glucose connected by α-1,4-bonds, and Dx is also a linear poly-D-glucose connected by α-1,6-bonds and possessing branches through α-1,3-bonds. It is also known that the average length of Dx branched chains is less than three glucose units [20–21]. Previously we have performed the mechanolysis of various types of polymers and found that the limiting molecular weight was more than 10,000 g/mol under our experimental conditions [5], thus the scission of Dx branched chains could not occur during mechanolysis. Instead, an α-1,6-glucosidic bond cleavage is expected to preferentially take place in the mechanolysis of Dx. As shown in Figure 4, four types of mechanoradicals could be produced by bond cleavage at α-1,4- and α-1,6-bond in each case. It has been reported that these end-chain radicals mechanically produced from polysaccharides, such as cellulose, HEC, amylose and so on, might be unstable at room temperature. Therefore these radicals could steeply abstract hydrogen from the surrounding glucose units to produce mid-chain alkyl radicals [5].

On the other hand, Gly is a hyperbranched poly-D-glucose connected through α-1,4-bonds with branches through α-1,6-bonds every 24 to 30 residues [22]. So, Gly mechanoradicals would be initially generated by α-1,4- and/or α-1,6-bond cleavage in the course of vibratory milling. Subsequently the mechanoradicals could undergo a following reaction, such as hydrogen abstraction to generate other types of radicals. Thus, the differences in the spectral patterns of amylose, Dx, and Gly could be due to the degree of hydrogen abstraction from the surrounding glucose units giving rise to glucose-derived mid-chain alkyl-type radicals and/or radical–radical coupling yielding non-radical species, followed by main-chain scission (Figure 4).

**Figure 5:**
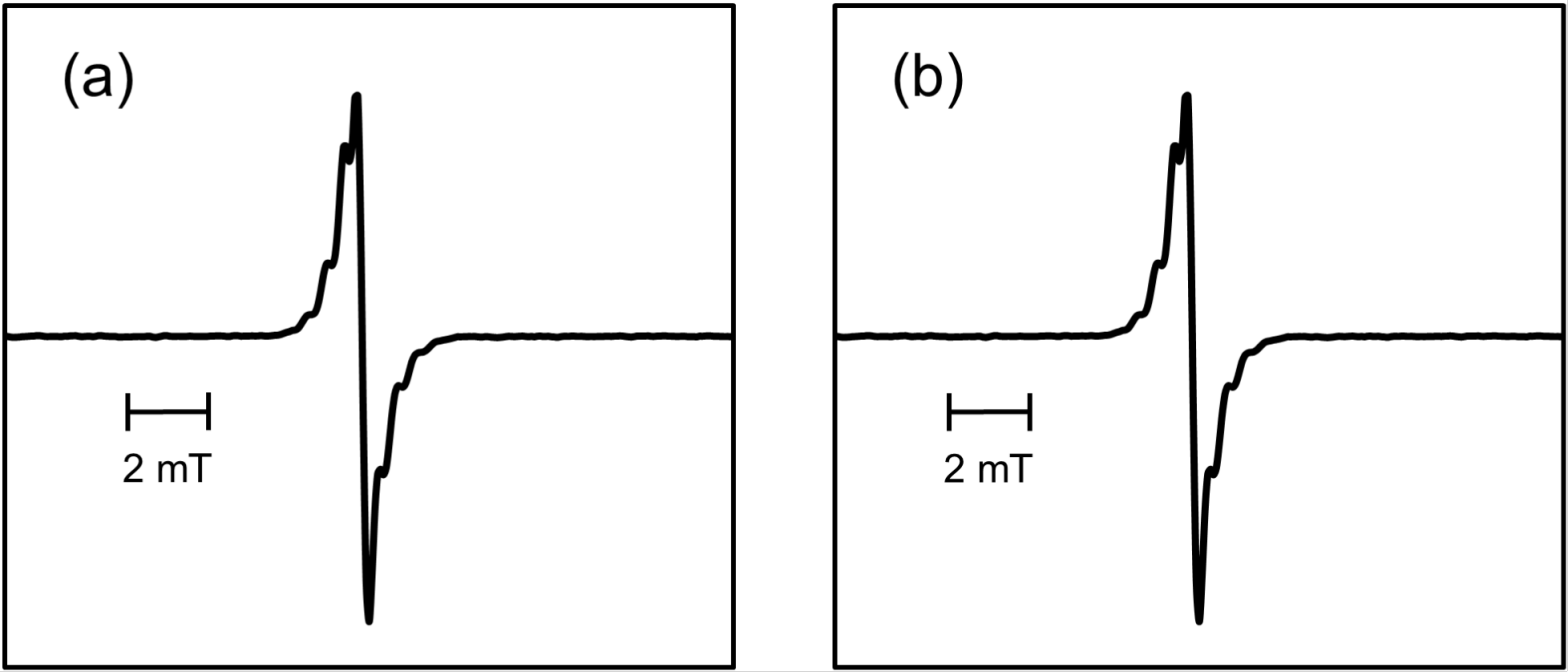
ESR spectrum of fractured sample of Dx and TCNE (a) before and (b) after visible-light irradiation.

Sakaguchi et al. reported that not only a homogeneous scission (mechanoradical formation) but also heterogeneous bond cleavage (mechanoanion formation) took place in the course of mechanochemical reaction of bacterial cellulose in a glass ball mill in vacuum in the dark at 77 K [23]. The same authors also demonstrated the modification of microcrystalline cellulose powder through mechanocation polymerization with isobutyl vinyl ether in vacuum at 77 K [24]. The aforementioned mechanoanion was confirmed through tetracyanoethylene (TCNE) radical anion formation. The latter radical is produced by a single-electron transfer from the mechanoanion to TCNE under visible-light irradiation. We adopted this method by Sakaguchi et al for the detection of mechanoanions (see Experimental). Figure 5 shows the observed ESR spectrum before and after visible-light irradiation of the fractured sample of Dx and TCNE.

As no ESR spectrum was observed after the mechanochemical reaction of pure TCNE, it was assumed that the ESR spectrum depicted in Figure 5a might be ascribed to the radical produced by the reaction of Dx mechanoradical and TCNE. As the characteristics of the spectrum and the intensity before and after visible-light irradiation remained unaffected, there was no mechanoanion in the fractured sample to a detectable extent. It was considered that a mechanoanion might promptly dissipate in the course of the mechanochemical reaction performed in a metallic vessel at room temperature.

To gain an insight into the component radicals a systematic computer simulation was performed for the ESR spectra of Dx and Gly and the results are shown in Figure 3 in an interrelated manner. The simulated spectra shown in Figure 3, represented as dotted lines, satisfactorily reproduced the observed.

**Figure 6:**
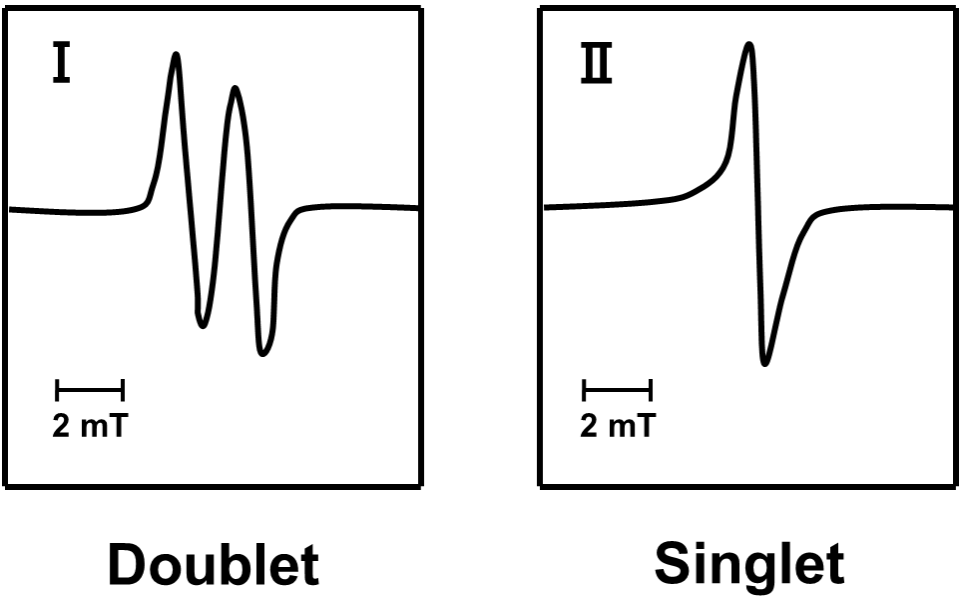
Component spectra of the simulated ESR spectra.

Figure 6 shows the spectral components of the simulated spectra: one doublet (I) and a singlet (II). The simulated spectra of Dx and Gly were obtained from I and II, similar to those of amylose [5]. In addition, all of the simulated spectra were reproduced with the different ratios of the component spectra.

The singlet spectrum (II) was the major component in the simulated Dx and Gly spectra and is assigned to a carbon-centered radical; an oxygen-centered radical has been excluded based on the *g*-value (ca. 2.0047 for Dx and Gly). This radical might have been formed through ring-opening and/or conjugating reactions after α-1,4- and/or α-1,6-glucosidic-bond cleavage and subsequent transformation and has no defined structure. On the other hand, we assigned the nearly isotropic doublet (I) to an alkoxylalkyl-type radical formed by hydrogen abstraction at the C1 position of the glucose unit, as assigned in the case of amylose. The ESR spectroscopic parameters for these Dx and Gly component spectra were consistent with those of amylose, and the associated parameters are shown in Table 1.

**Table 1:** ESR spectral data for component radicals in simulated spectra of amylose, Dx, and Gly.^a^

I	II	

	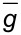 = 2.0047	*g*_1_ = 1.9999
*g* = 2.0052		*g*_2_ = 2.0067
		*g*_3_ = 2.0074
Aβ(1) = 1.70		

^a^HSC values are given in mT.

**Figure 7:**
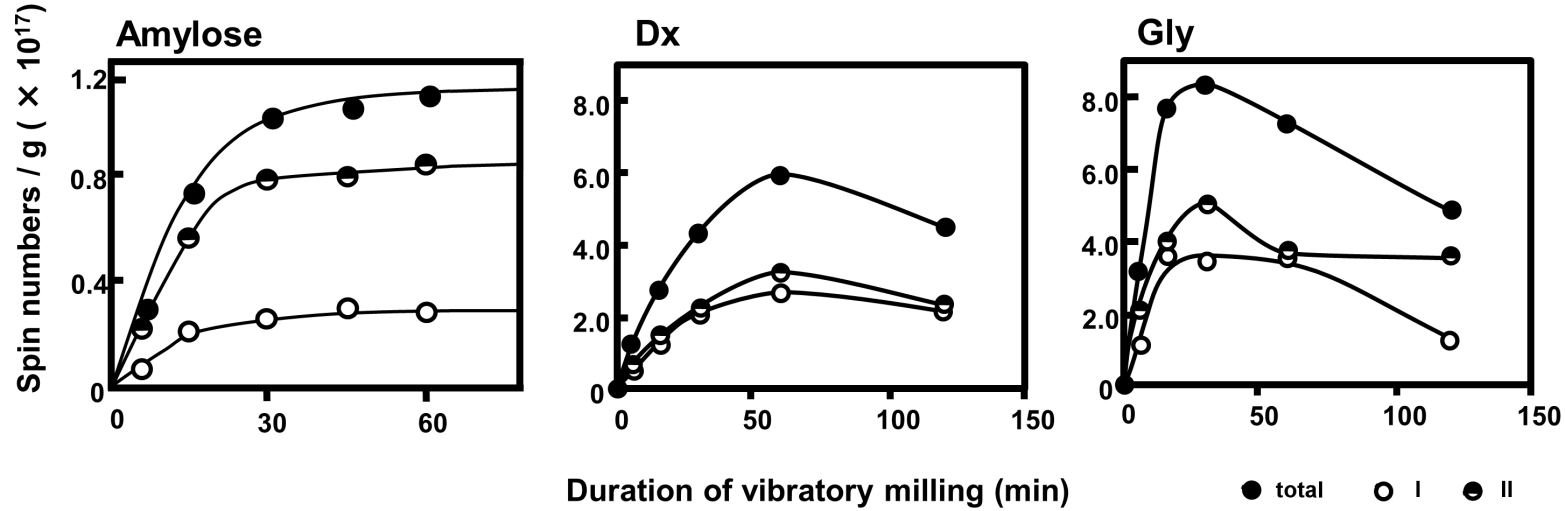
Progressive changes in the intensity of component spectra corresponding to the simulated spectra of amylose [5], Dx, and Gly.

The values for principal anisotropic parameters are only of semiquantitative significance, because these values slightly differed with the spectra. The progressive changes in the spectral intensity of the component radicals are shown in Figure 7, together with those of amylose for comparison.

For amylose, the total radical concentration did not decrease after 60 min of vibratory milling and also the ratio of amylose component radicals remained constant over time [5]. It was considered that the amylose mechanoradicals were more stable due to their intramolecular helical structure (rigid conformation). The maximum total radical concentration of Dx and Gly, however, was observed at 60 and 30 min, respectively. Afterwards the radical concentration gradually decreased. Kondo et al. and Solala et al. have also reported a similar behaviour of total radical concentration for polymethylmethacrylate and cotton, respectively [18,25]. These results suggested that the mechanoradicals produced during milling underwent radical–radical coupling and/or disproportionation reactions such as hydrogen abstraction to give non-radical species. We also reported the decrease of the total radical concentration in the mechanolysis of cellulose derivatives after achieving the maximum concentration. Here, an abstraction of hydrogen atoms from a substituted group of a cellulose derivative has been suggested and the resulting radicals disappeared rapidly due to radical recombination and/or disproportionation reactions [5]. As described above amylose has a rigid conformation due to a helical structure. On the other hand, Dx and Gly are more flexible due to their branched structures and it is assumed that the main and branched-chains of Dx and Gly move easier than the main-chain of amylose. This difference of the polymer structures might affect the elimination rate of mechanoradicals.

Furthermore, as shown in Figure 7, the spectral intensity ratio of each component radical of Dx did not change appreciably with the duration of vibratory milling. Although the spectral intensity of each Gly component radical increased within the first 30 min of reaction and gradually decreased thereafter, the progressive changes for the two components’ spectral intensity differed after 60 min. The spectral intensity of the doublet (I) assigned to an alkoxylalkyl-type radical decreased after 60 min, and that of the singlet (II) almost remained unchanged. The singlet (II) was assigned as a dangling bond site (DBS) that arose from ring-opened and/or conjugated polysaccharide structures. A DBS is a radical formed in a cross-linking region without defined structure (structureless). We compared the spectral intensities of the singlet (II) in Dx and Gly. In Dx the spectral intensity of II reached the maximum value at 60 min of vibratory milling, and then tended to decrease gradually. On the other hand, the spectral intensity of singlet II in Gly decreased after reaching the maximum value (30 min), but remained constant after 60 min. It was also shown that the spectral intensity of doublet I in Gly continued to decrease beyond 60 min, so that the total spectral intensity decreased. It was presumed that the DBS of Gly might be more stabilized than that of Dx due to higher cross-linking in the hyper-branched structure of Gly.

As mechanoradicals are formed by polymer main-chain scission [3–5], the quantity of mechanoradicals formed in the course of mechanolysis is associated with a change in molecular weight. To gain further insights into mechanoradical formation, we examined the progressive changes in the molecular weight of Dx using GPC analysis.

**Figure 8:**
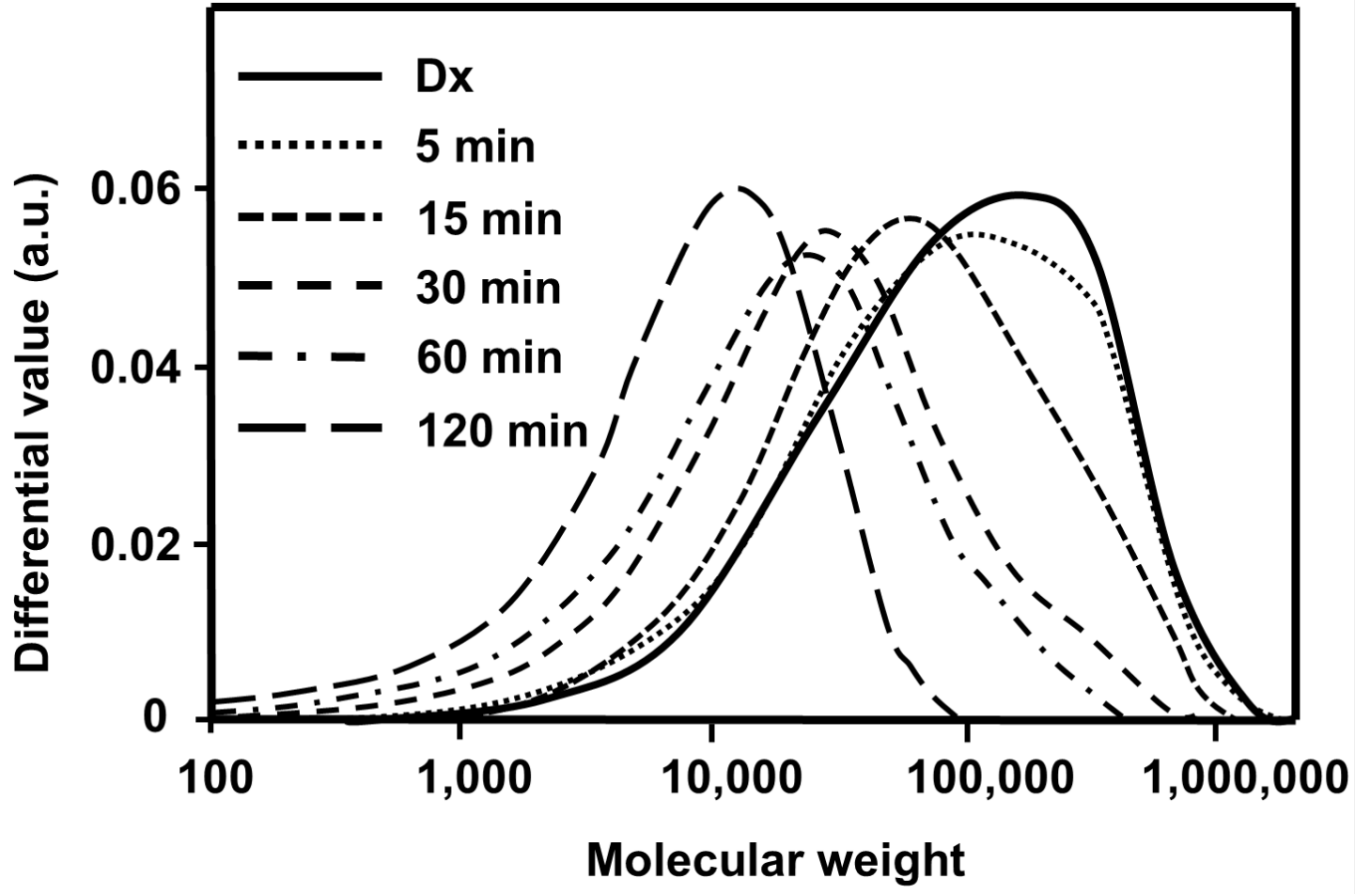
Changes in Dx molecular-weight distribution (MWD) during vibratory milling.

Figure 8 shows the changes in molecular-weight distribution (MWD) during the course of vibratory milling of Dx. A single broad MWD was observed regardless of milling duration, suggesting that polymer main-chain scission occurred randomly.

**Figure 9:**
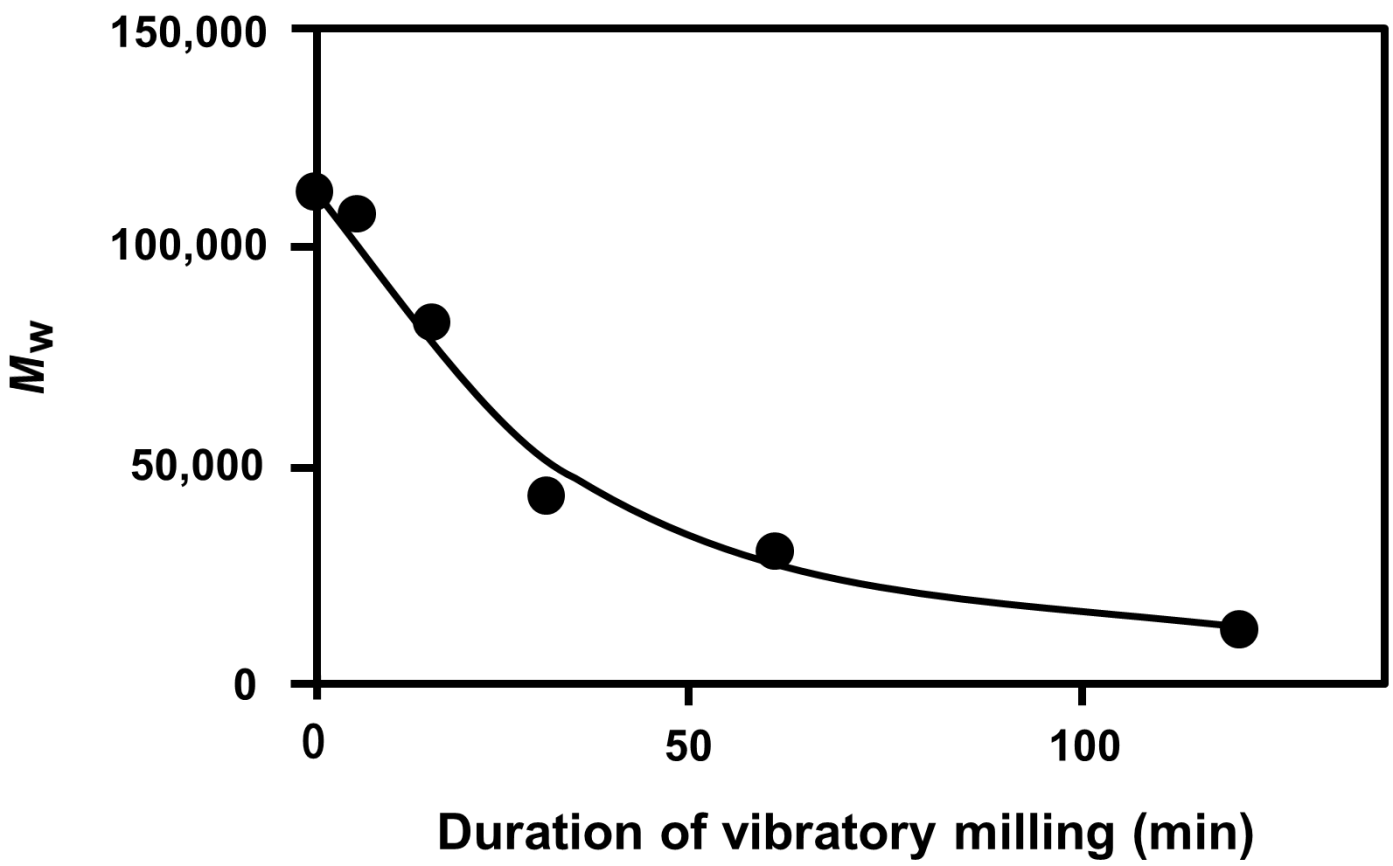
Changes in Dx weight-average molecular weight (*M*_w_) during vibratory milling.

The changes in the weight-average molecular weight (*M*_w_) over time of fractured Dx are shown in Figure 9.

As can be seen from Figure 9, the *M*_w_ of Dx decreased exponentially toward the limiting molecular weight (*M*_w,∞_) under the experimental conditions. As described above, the maximum Dx spectral intensity was observed at 60 min and the decrease in the molecular weight after 60 min was smaller than that before 60 min. This indicates that the mechanoradical formation is suppressed after 60 min. Thus, the changes in molecular weight are in good agreement with the change in radical concentration over time.

It is known from vibratory milling of several kinds of polymers that the *M*_w_ exponentially decreases toward *M*_w,∞_ which can be expressed as follows:

[1]
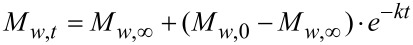


where *M*_w,t_ represents the molecular weight at a given mechanolysis time *t*, *M*_w,0_ indicates the molecular weight at *t* = 0, and *k* denotes the proportionality constant comprising system-dependent parameters [26–27]. The time-dependent changes in *M*_w_ depicted in Figure 9 fit the above Equation 1:

[2]
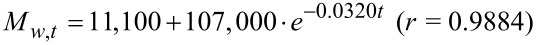


The *M*_w,∞_ of Dx was determined as 11,000 g/mol under the experimental conditions, similar to that of cellulose [5].

The concept of molecular weight is not suitable for a hyper-branched polysaccharide such as Gly. As Gly is a spherical polymer, it is considered that the particle diameter of Gly might decrease during the mechanolysis. Although a particle diameter of a hyper-branched polysaccharide could be measured by dynamic light scattering (DLS), it is difficult to precisely detect a particle with a diameter of less than 10 nm with our experimental setup.

**Figure 10:**
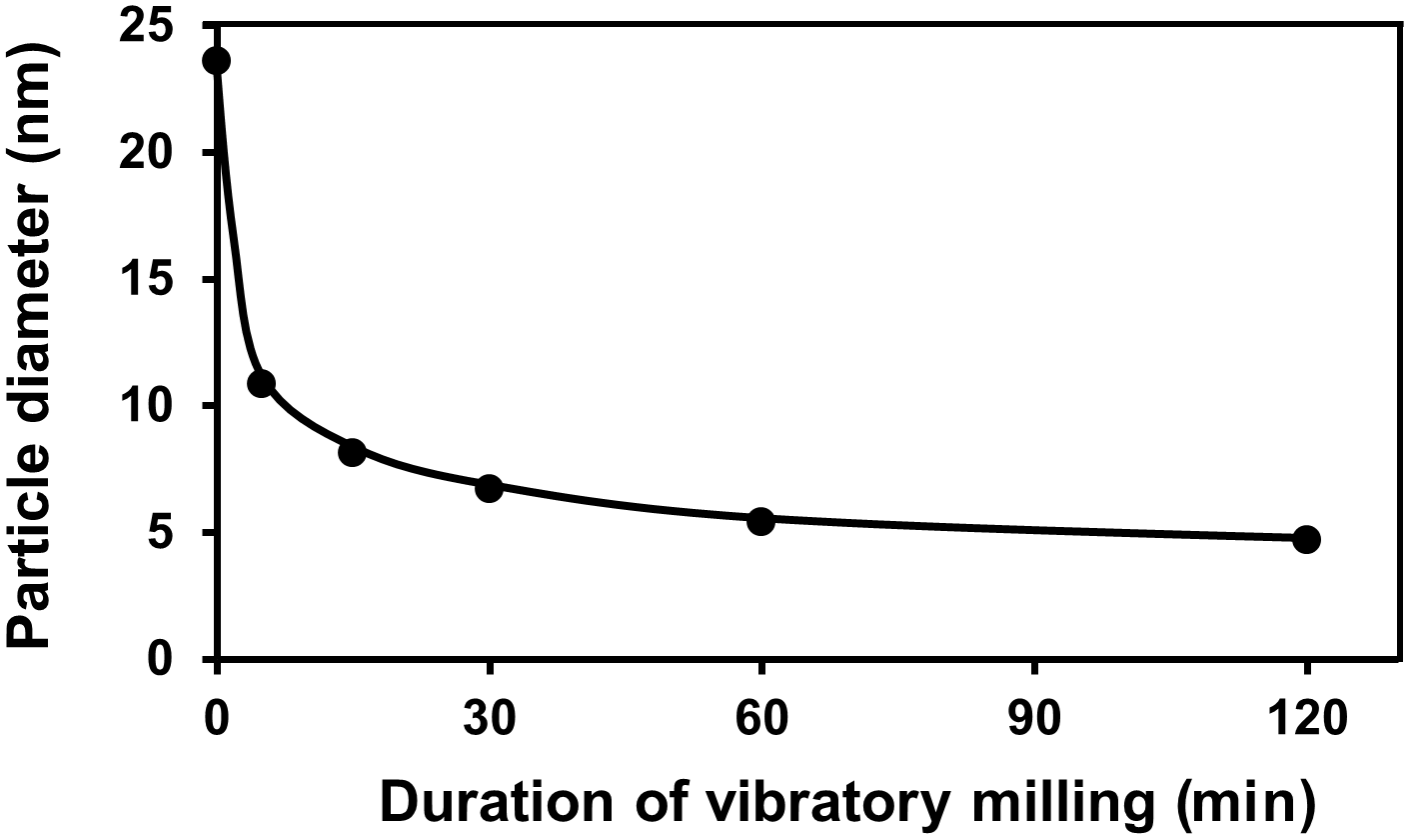
Change in Gly particle diameter during vibratory milling.

The hydrodynamic radius (*R*_h_) is utilized as an index of the spread of a polymer. It is well-known that the *R*_h_ of a spherical polymer dissolved in a solvent is correlated with its weight-average molecular weight. GPC is a size-exclusion technique in which molecules in solution are separated based on their size, and in some cases, based on their molecular weight. Pullulan, a linear polysaccharide, is a standard sample used in GPC analyses of polymers including polysaccharides. Rolland-Sabate et al. reported that the *R*_h_ of pullulan measured by DLS is proportional to the square of the weight average molecular weight determined by GPC [28]. Based on this result, we estimated the *R*_h_ of Gly by comparing its GPC elution time with that of pullulan as the standard. Figure 10 shows the time-dependent changes in the particle diameter (*R*_h_) of Gly during vibratory milling.

It can be seen from Figure 10 that the Gly particle diameter decreased rapidly up to 30 min of milling time and thereafter gradually decreased toward the limiting value. This result was consistent with the total radical concentration of Gly, which exhibited a maximum at 30 min (see Figure 7).

In both cases of Dx and Gly, the molecular weight of Dx or the particle diameter of Gly steeply decreased until reaching the maximum value of total radical concentration. Thereafter, the molecular weight or particle diameter gradually decreased toward the limiting value (Figure 9 and Figure 10). Thus, decreases in each component radical concentrations of Dx and Gly were due to intra- and/or intermolecular flexibility associated with their characteristic branched chains, different from amylose. Figure 7 also shows that the DBS in Gly was considerably more stable than that in Dx due to the higher cross-linking present in the hyper-branched structure of Gly.

## Conclusion

We discussed here the nature of mechanoradical formation during mechanolysis of Dx and Gly, based on ESR spectra coupled with systematic computer simulations, in comparison with the mechanolysis of amylose.

The component spectra of Dx and Gly were essentially identical to those of amylose and remained nearly unchanged in the course of vibratory milling. Simulated Dx, Gly, and amylose spectra were also obtained from admixtures of the component spectra at different ratios. Computer simulations revealed that a singlet spectrum (II) assignable to the immobilized DBS was a major component of milled Dx and Gly.

The generated Dx and Gly mechanoradicals dissipated more readily than amylose mechanoradicals in the course of vibratory milling. Amylose has a helical structure, and Dx and Gly exhibit branched structures and it was speculated that the difference of polymer structure among them could affect the dissipation of mechanoradicals. Thus, hydrogen atoms on the main and branched chains of Dx and Gly could be abstracted, so that the resulting mechanoradicals could rapidly disappear by radical recombination and/or disproportionation reactions due to the flexible structure. Additionally, the hyperbranched structure of Gly might be responsible for the greater stability of the DBS in Gly than that in Dx. The other component spectrum of milled Dx and Gly was a nearly isotropic doublet (I), which could be assigned to an alkoxylalkyl-type radical formed by hydrogen abstraction at the C1 position of the glucose unit, indicating the generation of glucose-based mid-chain alkyl-type radicals. The total radical concentration of both Dx and Gly decreased after reaching the maximum concentration, suggesting that the resulting mechanoradicals underwent radical–radical coupling and/or disproportionation reactions to produce non-radical species.

Systematic analyses of various physicochemical properties showed that the molecular weight of Dx and the particle diameter of Gly exponentially decreased toward the respective limiting value under the experimental conditions examined. This finding was consistent with the progressive changes in the respective radical concentrations. These results demonstrated that the quantity of the mechanoradicals generated during mechanolysis is correlated with the changes in molecular weight or particle diameter. The molecular weight of Dx and particle diameter of Gly approached to the limiting value after reaching the maximum value of total radical concentration (after 60 and 30 min for Dx and Gly, respectively). The disappearance of the Dx and Gly mechanoradicals began due to the presence of flexible branched chains. This phenomenon differed from the case of amylose, which possesses a helical intramolecular structure. The DBS in Gly was found to be more stable than that in Dx due to its hyperbranched structure. The present results also indicated that the mechanolysis of Dx at room temperature not only afforded lower molecular weight polymers but also led to partial decomposition of the Dx structure by ring-opening and/or conjugating reaction to emerge the cross-linking region. If one performs the mechanolysis of Dx open to air, such structural decomposition of Dx might have occurred and some oxidized functional groups could be incorporated in Dx. The present findings are expected to facilitate graft polymerization of vinyl or acryl monomers onto Dx and Gly.

## Experimental

### Materials

Powdered Dx (clinical grade), was purchased from Wako Co., Ltd., passed through a 200–235 mesh sieve, and then dried at 60 °C for 12 h in vacuo. Powdered Gly (from Oyster, reagent for molecular biology) was purchased from Nacalai Tesque Co., Ltd. and treated in a similar way to Dx.

### Mechanolysis methods

Analogous to the description in [19], powdered samples (100 mg) were mechanically fractured under a nitrogen atmosphere in a vibratory ball-milling apparatus (Shofu Co., Ltd., Kyoto, Japan) equipped with a stainless steel twin-shell blender (7.8 mm diameter, 24 mm length) and a stainless steel ball (6.0 mm diameter, 890 mg) at room temperature for a prescribed period of time at 60 Hz. Residual oxygen was removed using a Model 1000 Oxygen Trap (Chromatography Research Supplies Inc., Louisville, US) and the oxygen concentration was monitored using an oxygen analyser (LC750/PC-120, Toray Engineering Co., Ltd., Shiga, Japan) and kept below 0.01 ppm. The fractured samples were transferred to an ESR tube, which was then sealed and subjected to ESR analysis. All sample manipulations were carried out in a vacuum glove box (Sanplatec Corp., Osaka, Japan). The mechanolysis was carried out for the experimental time points to obtain the fractured sample.

### ESR spectral measurements

Similarly as described in [19], ESR spectra were recorded on a JES-RE1X (JEOL Ltd., Japan) spectrometer with X-band and 100 kHz field modulation. Special care was taken to ensure that no saturation occurred and that the line shape was not distorted by excessive modulation amplitude. The square root of the microwave power versus the signal peak height was plotted, so that a microwave power level of 0.04 mW was chosen. The ESR spectral intensity was determined by double integration. The radical concentration (spin numbers per gram of sample) was calculated from the spectral intensity of a poly(methyl methacrylate) sample and impregnating with 2,2-diphenyl-picrylhydrazyl. ESR spectra were measured for all experimental time points. The observed ESR spectra were unchanged for at least several hours at room temperature in the intensity and shape within a detectable extent.

### Procedure to detect mechanoanions

Dx was fractured in a metallic vessel at room temperature. The fractured Dx and TCNE were mixed in the dark to avoid the decomposition of mechanoanion and exposed to visible light to induce electron release. After vigorously shaking of the mixture it was transferred to an ESR tube in the dark. ESR spectra were taken before and after visible-light irradiation.

### Molecular weight measurements

Similarly as described in [19], the molecular weight of each resulting polymer was measured by gel-permeation chromatography (GPC) using a PU 610 HPLC pump (GL Sciences Inc., Tokyo, Japan) equipped with an RI 504R refractive index detector (GL Sciences Inc.), a model 556 LC column oven (GL Sciences Inc.), gel column (GF-1G 7B and GF-7M HQ, Shodex, Kawasaki, Japan), and a data analyser (Runtime Instruments Chromato-PRO, Runtime Instruments Ltd., Tokyo, Japan). The following conditions were applied: elution solvent, distilled water containing 0.05 wt/vol % NaCl; flow rate, 0.7 mL/min; column temperature, 40 °C. Calibration was carried out with pullulan standards (peak top molecular weight [*M*_peak_] = 5,900, 9,600, 21,100, 47,100, 109,000, 200,000, 344,000, and 708,000 g/mol).

### Dynamic light scattering measurements

Analogous to the description in [29], dynamic light scattering was measured using a DLS-5500G Photal dynamic light scattering spectrophotometer (Otsuka Electronics Co., Ltd., Osaka, Japan) equipped with a He/Ne laser. A scattering angle of 90° was used in this study. The hydrodynamic diameter and the polydispersity factor of the polymers, represented as μ_2_/Γ^2^, were calculated using the Stokes–Einstein equation and the cumulant method. The number-average particle diameter and weight-average particle diameter were determined by the histogram method with the Marquardt calculation.

### Computer simulations of ESR spectra

Analogous to the description in [5], computational simulations were performed on a personal computer (DELL Inspiron 545S) using a simulation program developed in our laboratory. The simulated spectra were obtained from Lorentzian functions by iterative fitting of the spectroscopic parameters (*g-*value, line width at half-height, hyperfine splitting constant [HSC], and relative intensity) with the observed digitized spectra using a non-linear least-squares method [30–36]. The simulation program included the effect of anisotropy in the *g*-factor and/or α-hydrogen hyperfine tensor on the line shape of powder spectra, according to Kneubuhl’s [37] and Cochran’s [38] equations, respectively. An anisotropic interaction of β-hydrogens is usually small (less than 0.3 mT), so that such an effect is easily blurred due to broadening of the width of the individual peak and was therefore not considered in the spectral simulations. To assist the simulation procedure, we also enhanced the program for obtaining the difference spectra by subtracting one observed spectrum from another.
